# Oncogenic features of the bone morphogenic protein 7 (BMP7) in pheochromocytoma

**DOI:** 10.18632/oncotarget.4912

**Published:** 2015-08-18

**Authors:** Ines Leinhäuser, Andrea Richter, Misu Lee, Ines Höfig, Nataša Anastasov, Falko Fend, Tonino Ercolino, Massimo Mannelli, Anne-Paule Gimenez-Roqueplo, Mercedes Robledo, Ronald de Krijger, Felix Beuschlein, Michael J. Atkinson, Natalia S. Pellegata

**Affiliations:** ^1^ Institute of Pathology, Helmholtz Zentrum München, Neuherberg, Germany; ^2^ Institute of Radiation Biology, Helmholtz Zentrum München, Neuherberg, Germany; ^3^ Institute of Pathology and Neuropathology Comprehensive Cancer Center Tübingen and University of Tübingen, Tübingen, Germany; ^4^ Azienda Ospedaliero-Universitaria di Careggi, Endocrine Unit, Florence, Italy; ^5^ Department of Experimental and Clinical Biomedical Sciences, University of Florence, Florence, Italy; ^6^ INSERM, UMR U970, Paris Cardiovascular Research Center-PARCC, Paris, France; ^7^ Université Paris Descartes, Sorbonne Paris Cité, Faculté de Médecine, Paris, France; ^8^ Assistance Publique Hôpitaux de Paris, Hôpital européen Georges Pompidou, Department of Genetics, Paris, France; ^9^ Hereditary Endocrine Cancer Group, Spanish National Cancer Research Centre (CNIO), Madrid, Spain; ^10^ Department of Pathology, Erasmus MC, University Medical Center Rotterdam, Rotterdam, The Netherlands; ^11^ Endocrine Research Unit, Medizinische Klinik und Poliklinik IV, Klinikum der Universität München, München, Germany

**Keywords:** pheochromocytoma, bone morphogenic protein 7, PI3K pathway, integrin beta 1, MENX rats

## Abstract

BMP7 is a growth factor playing pro- or anti-oncogenic roles in cancer in a cell type-dependent manner. We previously reported that the *BMP7* gene is overexpressed in pheochromocytomas (PCCs) developing in MENX-affected rats and human patients. Here, analyzing a large cohort of PCC patients, we found that 72% of cases showed elevated levels of the BMP7 protein. To elucidate the role of *BMP7* in PCC, we modulated its levels in PCC cell lines (overexpression in PC12, knockdown in MPC and MTT cells) and conducted functional assays. Active BMP signaling promoted cell proliferation, migration, and invasion, and sustained survival of MENX rat primary PCC cells. In PCC, BMP7 signals through the PI3K/AKT/mTOR pathway and causes integrin β1 up-regulation. Silencing integrin β1 in PC12 cells suppressed BMP7-mediated oncogenic features. Treatment of MTT cells with DMH1, a novel BMP antagonist, suppressed proliferation and migration. To verify the clinical applicability of our findings, we evaluated a dual PI3K/mTOR inhibitor (NVP-BEZ235) in MENX-affected rats *in vivo*. PCCs treated with NVP-BEZ235 had decreased proliferation and integrin β1 levels, and higher apoptosis. Altogether, BMP7 activates pro-oncogenic pathways in PCC. Downstream effectors of BMP7-mediated signaling may represent novel targets for treating progressive/inoperable PCC, still orphan of effective therapy.

## INTRODUCTION

Pheochromocytomas (PCCs) are tumors arising from neural crest-derived chromaffin cells of the adrenal medulla and sympathetic ganglia (the latter referred to as paragangliomas, PGLs). PCCs/PGLs occur sporadically or as a result of an inherited germline mutation in one of at least 12 genes (35%–40% of cases), including *VHL*, *NF1*, *RET*, *SDH*A, *SDHB*, *SDHC*, *SDHD*, *SDHAF2*, *HIF2α*, *KIF1Bβ*, *TMEM127, MAX* and *FH* [1]. In contrast to familial PCC/PGL, less is known about the somatic mechanisms driving the more frequent sporadic tumors. Recently, using an integrative genomics approach, common alterations were discovered in sporadic PCC that now await functional validation [2, 3].

Transcriptome analyzes have determined that gene expression signatures of human PCCs reflect the underlying driver mutation [4,5]. Specifically, PCCs and PGLs can be divided into two main clusters, designated as Cluster 1 and Cluster 2; Cluster 1 tumors are associated with germline mutations in *VHL*, *SDHx*, and probably *HIF2α* and *FH* genes, and Cluster 2 tumors are associated with mutations in *NF1*, *RET*, *KIF1Bβ*, and *TMEM127* [1]. Sporadic PCCs mainly group in Cluster 2.

Although usually benign, approximately 10–15% of PCC cases are considered malignant on the basis of the presence of distant metastases and have a 5-year survival rate of <50% [6]. Surgery remains the first-line therapy for patients with localized disease or with isolated and resectable distant metastases [7]. For patients with disseminated tumor spread, extensive local invasion, or recurrence, systemic conventional chemotherapy has been tested without clear benefit on overall survival. Radiotherapy with the radiopharmaceutical ^131^I-meta-iodobenzylguanidine (^131^I-MIBG) was shown to have positive therapeutic effects, but tumor regression occurred in only 30% of patients [8]. The tyrosine kinase inhibitor sunitinib has shown some efficacy in patients with a progressive disease [9], whereas the mTOR inhibitor everolimus exhibited low efficacy [10, 11]. Taken together, there is a considerable clinical need for more effective therapies against aggressive/malignant PCC; elucidating the molecular mechanisms involved in PCC tumorigenesis will be instrumental in identifying targets for such therapies.

Rats affected by the MENX multiple endocrine neoplasia syndrome develop bilateral PCCs with complete penetrance [12]. The tumors show progression from hyperplasia (4 months of age) to tumors (7–8 months of age) with time. Rat PCCs share similarities with their human counterparts in terms of histopathological features [13, 14], gene copy number variations [13], expression signatures [14], and uptake of radiolabeled tracers for imaging [15, 16]. The rat tumors show elevated proliferation rates (average 11.3%, range 3.7% to 16.7%) [15], thus mostly resembling human aggressive PCCs. Despite these high proliferation rates, no metastases of rat pheochromocytomas have been so far documented, probably due to the short life span of MENX rats [17]. To elucidate the molecular pathogenesis of PCC, we previously performed transcriptome analyzes of adrenomedullary hyperplasia and tumors from MENX-affected rats. These studies identified the *Bmp7* gene, never before associated with adrenomedullary tumorigenesis, as being significantly overexpressed in rat hyperplastic and neoplastic lesions *versus* normal adrenal medulla [14]. *Bmp7* was already up-regulated in the adrenal medulla of 1-month-old mutant rats before pathological changes occur. Importantly, the *BMP7* gene was also found to be up-regulated in 88% of human sporadic PCCs and 69% of the familial cases [14].

BMP7 (bone morphogenic protein 7) belongs to the transforming growth factor β (TGFβ) superfamily of secreted growth factors [18]. Besides a role in embryonic development, differentiation, and organogenesis, BMPs were recently implicated in regulating growth, migration, and apoptosis of cancer cells [19–21]. BMPs bind to types I and II transmembrane serin/threonine kinase receptors (BMPR-I or BMPR-II), which dimerize upon ligand binding, and the constitutively activated BMPR-II phosphorylates BMPR-I [22]. In the canonical BMP pathway, BMPRI phosphorylates receptor-associated SMAD transcription factors [SMADs 1, 5, and 8 (mouse)/9 (human)], which then bind to the common mediator SMAD4 and translocate into the nucleus [22], where these complexes bind to the DNA and regulate target gene transcription [23].

In human cancers, BMPs exhibit pro- or anti-carcinogenic functions depending on the cell/tissue type, dosage, and micro-environment [24]. Although the functional significance of BMP7 has been studied in several tumors, including breast, colorectal, gastric, and prostate carcinomas [25–28], its role in adrenomedullary tumorigenesis has not yet been explored. Using a large cohort of well-characterized PCC/PGL patients, we here confirmed that the BMP7 protein is highly expressed in >70% of tumors; this correlates with extra-adrenal location and tumor size. We demonstrated that endogenous *BMP7* overexpression promotes PCC cell proliferation, migration, and invasion and identified the PI3K/AKT/mTOR pathway and integrin β1 as downstream effectors of active BMP signaling. Proof-of-principle studies using a specific BMP antagonist (*in vitro*) and a PI3K/mTOR inhibitor (*in vivo*) revealed that targeting BMP7-mediated pathways could be an effective strategy to treat PCCs.

## RESULTS

### Bmp7 expression in rat PCCs

**Figure 1:**
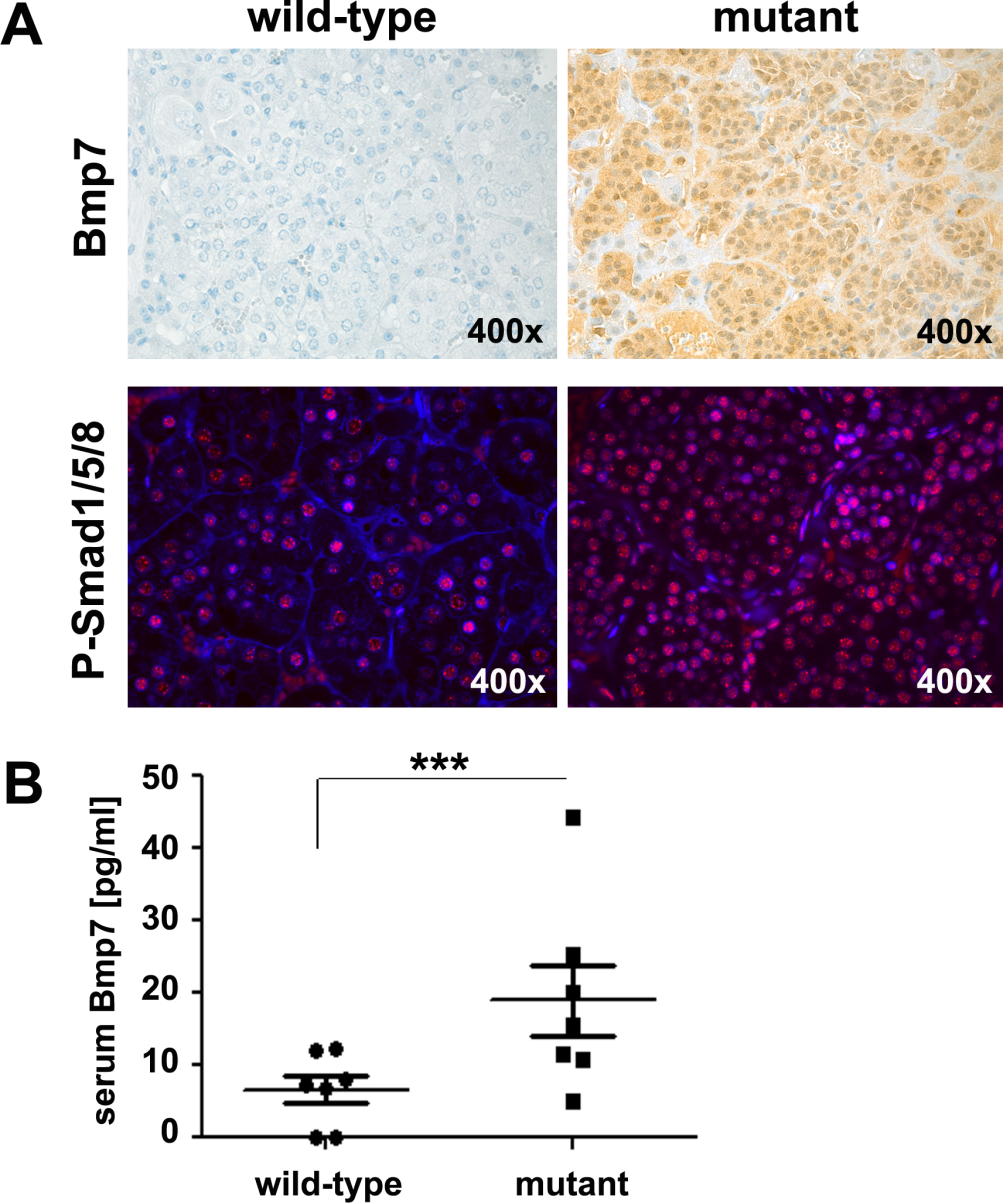
Expression of Bmp7 in rat PCC and its secretion **A.** IHC or IF on adrenal medullary tissue from wild-type and mutant rats was performed using a rat anti-BMP7 antibody or an anti-P-Smad1/5/8 antibody, respectively. These tissues are representative of five tissues per animal group. Original magnification: 400× **B.** Plasma Bmp7 levels were measured in seven MENX mutant rats and seven wild-type rats fasted for 12 h. Measurements were performed using a rat Bmp7 ELISA-KIT. ***, *P* < 0.001.

We investigated whether *Bmp7* transcript overexpression, previously observed in MENX-associated rat PCCs [14], translates into increased levels of the encoded protein. By immunohistochemistry (IHC) and immunofluorescence (IF), we could confirm that Bmp7 protein levels and those of its canonical downstream targets [i.e., phosphorylated (P)-Smad1/5/8] were elevated in the tumors of mutant animals compared with the adrenal medulla of wild-type animals (Figure 1A). Concordantly, circulating Bmp7 protein was significantly elevated in the peripheral blood of mutant when compared with wild-type animals (*P* = 0.0006) (Figure 1B). Collectively, these data demonstrate that the Bmp7 protein is expressed at a high level in rat PCCs and is secreted.

### BMP7 expression in human PCCs

**Figure 2:**
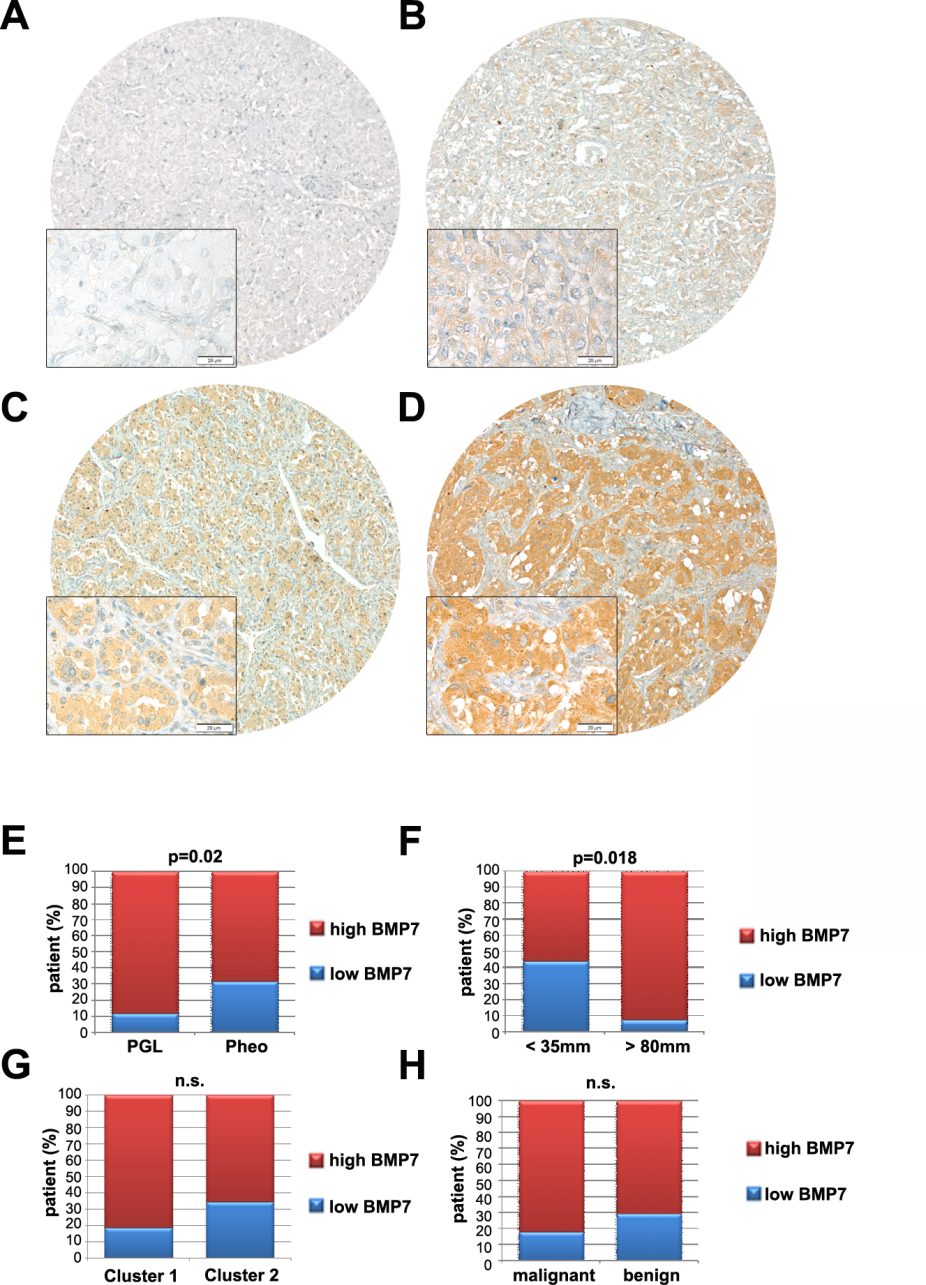
Expression of BMP7 in human PCC **A–D.** IHC for BMP7 was performed on tissue microarrays using a human anti-BMP7 antibody. After selection, 150 PCCs and 34 PGLs were scored. We only considered tumors for which both cores could be scored after IHC staining. Images were recorded and we show low (original magnification, × 40) and high (original magnification, × 400) power views of four different PCCs representative of the intensities of BMP7 staining (A,−; B,+; C,++; D,+++). **E–H.** BMP7 expression of the 184 tumors was correlated with clinical variables, including (E) location (Extra-adrenal PCC = PGL; Adrenal PCC = Pheo), (F) tumor size (<35 mm; >8 mm), (G) genetic background (cluster 1; cluster2), and (H) malignancy (malignant; benign). n.s., not significant.

Similar to the rat tumors, human sporadic and familial PCCs also overexpress the *BMP7* gene [14]. Following preliminary immunohistochemical studies on 10 human primary PCCs, we extended our analysis to a large and well-characterized cohort of 208 PCCs/PGLs spotted on tissue microarrays (TMAs). A total of 184 tumor samples (150 PCCs and 34 PGLs) satisfied our inclusion criteria (see Material and Methods). IHC was performed and TMAs were scored for staining intensity. The anti-BMP7 antibody we used has a reported 25% cross-reactivity with BMP6 and no cross-reactivity with other BMPs. However, given that the *BMP6* gene was found to be expressed at the limit of detection in a series of 10 frozen human PCCs by TaqMan (data not shown), the signal observed by IHC is specific for BMP7. In our cohort, 72% of the tumors displayed moderate (++) to strong (+++) immunoreactivity for BMP7 (Figure 2A–2D). We then studied the possible correlations between BMP7 levels and either clinical parameters of the patients or tumor characteristics. No association between BMP7 expression and gender, age, plasma catecholamines, or chromogranin A levels was evident. In contrast, we found that 88% of the PGLs, but only 68% of PCCs, exhibit an elevated BMP7 expression (*P* = 0.02, Figure 2E). BMP7 levels were high (++/+++) in 93% of tumors bigger than 8 cm in diameter (large), but only in 56% of the tumors smaller than 3 cm in diameter (small, *P* = 0.018, Figure 2F). Concerning the underlying predisposing mutation, we found that 92% of Cluster-1 tumors show high BMP7 levels as opposed to 74% of Cluster-2 tumors; however, this difference was not statistically significant (Figure 2G). In our series, 11 cases were malignant and nine of them showed high BMP7 expression, suggesting a trend for higher BMP7 levels in malignant tumors (Figure 2H).

Thus, in human PCCs, BMP7 is highly expressed and its levels positively associate with tumor size and origin (extra-adrenal).

### BMP7 promotes proliferation of PCC cell lines and the viability of primary rat PCC cells

**Figure 3:**
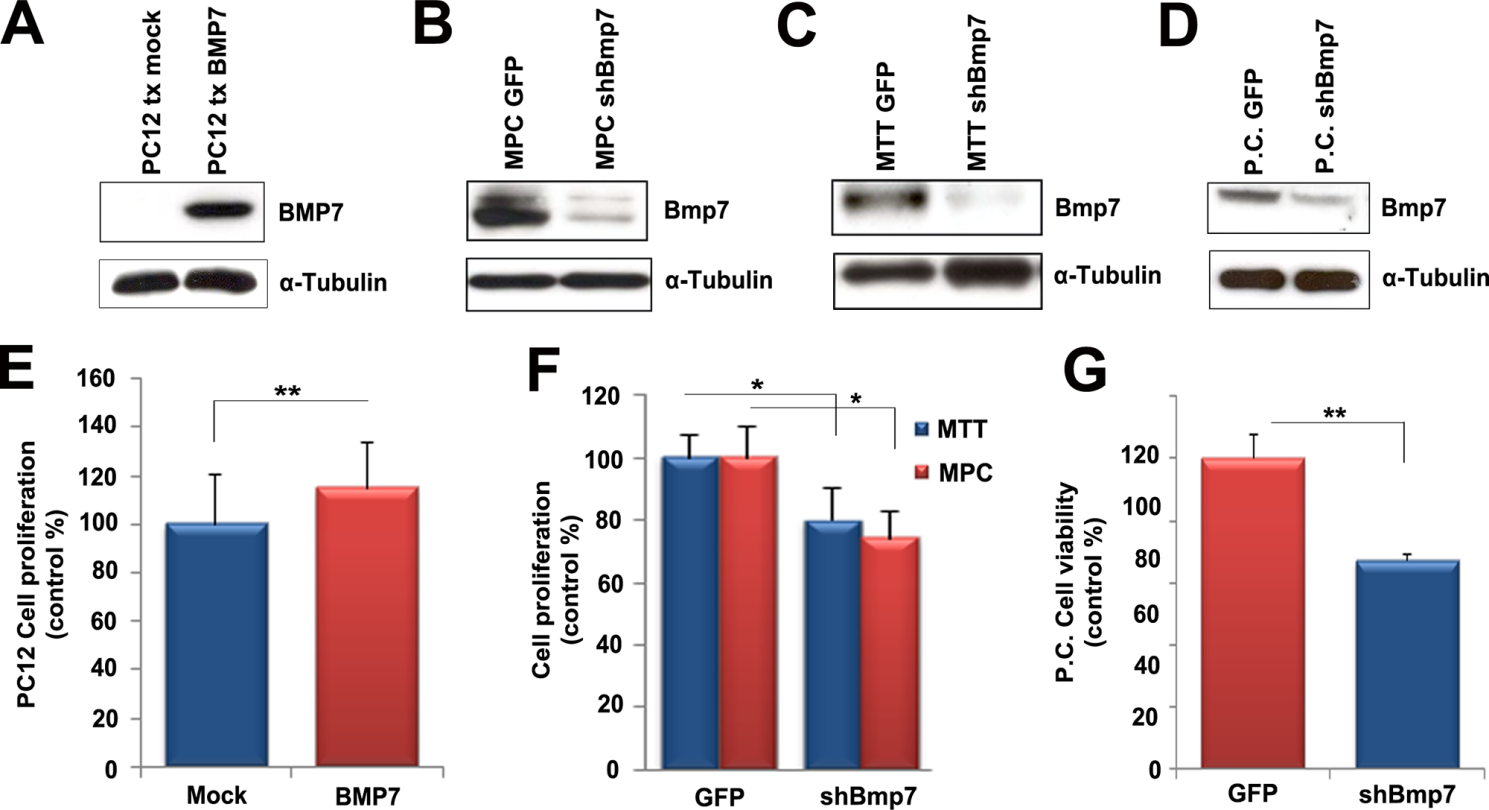
Bmp7 promotes proliferation of PCC cells *in vitro* **A.** We transfected PC12 cells with a Myc-BMP7 plasmid, and 24 h later cells were analyzed by western blotting using a specific anti-Myc antibody. **B.** MPC, **C.** MTT and **D.** primary rat tumor (P.C.) cells were infected with lentiviral vectors containing shBMP7-GFP (#2.9) or GFP alone. Western blotting was performed 72 h later using the rat anti-BMP7 antibody. α-Tubulin was used as loading control. **E.** PC12 cells were transfected with the BMP7 plasmid (BMP7) or with the mock vector (Mock) and proliferation was assessed 24 h later using the WST-1 assay. Data were analyzed independently with six technical replicates each, and are expressed as the mean ± SD. Proliferation was normalized against the values of mock transfected cells arbitrarily set to 100%. **, *P* < 0.01. **F.** MPC and MTT cells were infected as in B and C, and 72 h later we assessed proliferation using the WST-1 assay. Data were analyzed independently with six technical replicates each, and are expressed as the mean ± SD. Proliferation was normalized against the values of GFP-infected cells arbitrarily set to 100%. *, *P* < 0.05. **G.** Primary cells (P.C.) were infected as in D and cell viability was measured 72 h after infection. The average of three independent cultures from three mutant rats (8 months of age) is shown. Proliferation was normalized against the values of GFP-infected cells which were arbitrarily set to 100%. **, *P* < 0.01.

As no data were available on the possible role of Bmp7 in PCC, we performed *in vitro* functional assays. We first employed as experimental model the PC12 cell line, established from a rat PCC [29]. Consistent with published data [30], PC12 cells were found to express BMP receptors, thereby representing a suitable model to study autocrine/paracrine effects of endogenous BMP signaling (Supplementary Figure S1). PC12 cells express the endogenous *Bmp7* gene at relatively low levels (Figure 3A). Thus, they were transfected with an empty vector (mock) or with a vector expressing a Myc-tagged human *BMP7* cDNA to mirror the situation in rat and human primary PCCs (Figure 3A). Ectopic *BMP7* overexpression slightly increased PC12 proliferation rates (*P* < 0.01) (Figure 3E).

MPC and MTT mouse PCC cell lines were found to have relatively high endogenous Bmp7 levels (Figure 3B, 3C) and were therefore utilized for knockdown experiments. Similar to PC12 cells, MPC and MTT cells were found to express BMP receptors (Supplementary Figure S1). Because these cells were difficult to transfect, we generated lentiviral vectors expressing small hairpin (sh)RNA molecules directed against both mouse and rat *Bmp7* gene together with the green fluorescent protein (GFP) to monitor infection efficiency (around 80%–90%, data not shown). Two sequences (#1.8 and #2.9) were originally cloned and tested in preliminary experiments that showed that GFP vector alone had no effect on cell proliferation, while both sh*Bmp7* constructs decreased cell proliferation (Supplementary Figure S2A). Since sequence #2.9 reduced *Bmp7* expression more efficiently (Supplementary Figure S2B), it was selected for further experiments.

We found that reduced endogenous *Bmp7* expression decreased the proliferation of MPC (30% reduction; *P* < 0.05) and MTT (20% reduction; *P* < 0.05) cells (Figure 3F). *Bmp7* knockdown in sh*Bmp7*-infected cells was confirmed by western blotting (Figure 3B, 3C). We then evaluated the potential tumorigenic role of Bmp7 in a more physiological system: primary cultures of MENX-derived PCCs. We first confirmed that the tumor cells *in vitro* maintain the high Bmp7 expression seen in the primary tumors of origin (Figure 3D) and that they express BMP receptors (Supplementary Figure S1). Preliminary experiments showed that sh*Bmp7* #2.9 lentiviral vector was the most effective vector also in this experimental system and was further employed (Supplementary Figure S2C, S2D). Then, PCC cells from three mutant rats were dispersed in culture plates and infected with sh*Bmp7*-GFP or GFP-only lentiviral vectors. Concomitantly, with a reduction of *Bmp7* expression (Figure 3D), we observed a significant decrease in primary cell viability upon infection with sh*Bmp7-*vector (35%–45% reduction *versus* control GFP-vector) (Figure 3G). Therefore, autocrine *Bmp7* signaling plays a role in sustaining the survival of rat primary adrenomedullary tumor cells.

### BMP7 enhances migration and invasion of PCC cells

**Figure 4:**
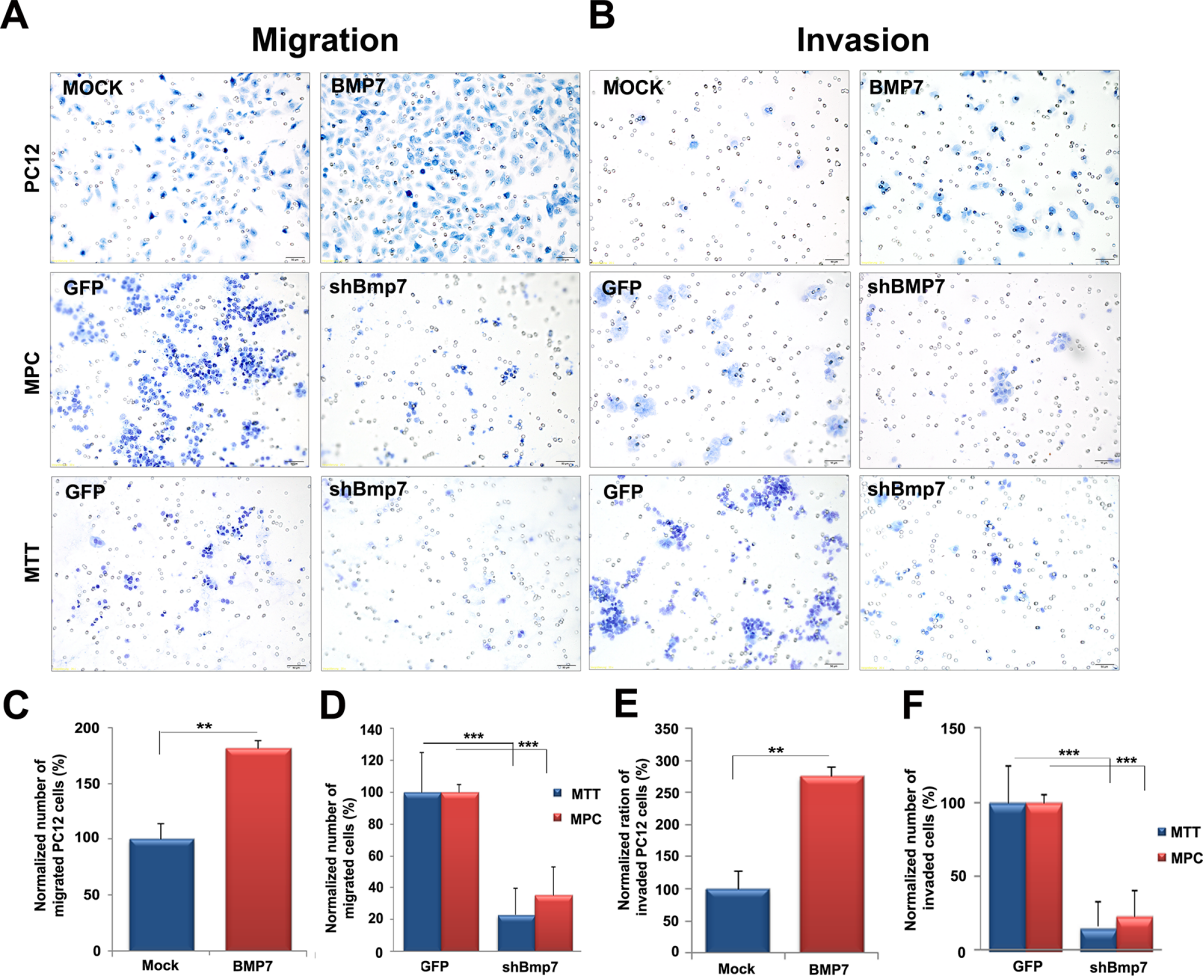
Bmp7 enhances migration and invasion of PCC cells *in vitro* We transfected PC12 cells with a Myc-BMP7 plasmid and 24 h later **A, C.** migration and **B, E.** invasion were assessed. MPC and MTT cells were infected with lentiviral vectors containing sh*Bmp7*-GFP or GFP alone, and 72 h later we assessed **A, D.** migration and **B, F.** invasion. The percentage of cells that migrated or invaded was normalized against the values of mock transfected cells or of GFP-infected cells arbitrarily set to 100%. The experiment was performed two times with three technical replicates with similar results. Five random fields of each test at ×400 magnification were counted (±SD). **, *P* < 0.01; ***, *P* < 0.001.

Next, we investigated whether BMP7 promotes cell migration and/or invasion of PCC cells. BMP7- or empty-vector-transfected PC12 cells were used for migration assays employing a Boyden chamber. Elevated *BMP7* expression stimulated PC12 cell migration (1.8-fold that of empty vector-transfected cells) (Figure 4A, 4C). Invasion assays through a Matrigel basement membrane matrix were conducted in parallel. BMP7-transfected PC12 cells displayed higher invasion potential (2.8-fold) than empty-vector-transfected cells (*P* < 0.01) (Figure 4B, 4E). Similar experiments conducted on MPC cells showed that *Bmp7* downregulation by the sh*Bmp7*-containing vector reduced their migration and invasion potential by up to 65% (*P* < 0.001) and 70% (*P* < 0.001), respectively, compared with GFP-vector-infected cells (Figure 4A, 4B, 4D, 4F). Similarly, migration (average 70% reduction; *P* < 0.001) and invasion (80%; *P* < 0.001) of MTT cells were reduced upon *Bmp7* knockdown (Figure 4A, 4B, 4D, 4F).

Thus, high endogenous Bmp7 levels promote proliferation, but especially migration and invasion (i.e., the metastatic potential) of PCC cells.

### BMP7 pathway activation increases integrin β1 expression

**Figure 5:**
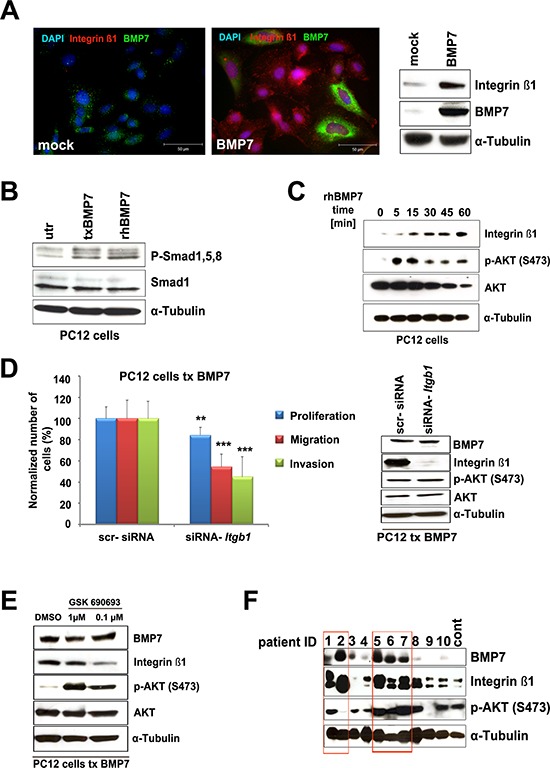
Downstream BMP7 signaling in PCC cells **A.** PC12 cells were transfected with a BMP7-containing (BMP7) or an empty (mock) vector. Twenty-four h post transfection IF was performed using specific antibodies targeting integrin β1 (1:400) or BMP7 (1:100). Cell nuclei were counterstained with DAPI. In parallel, the expression of integrin β1 and BMP7 proteins was determined by western blotting using specific antibodies. α-Tubulin was used as loading control. **B.** PC12 cells were either transfected (txBMP7) with a Myc-BMP7 plasmid or treated with 100 ng/mL recombinant human BMP7 (rhBMP7). After 24 h, cells were collected and analyzed by western blotting using antibodies against P-Smad1/5/8 and Smad1. α-Tubulin was used as a loading control. **C.** PC12 cells were treated with rhBMP7 for 5, 15, 30, 45, or 60 min. Western blot analysis was performed using specific antibodies raised against integrin β1, AKT and P-AKT. α-Tubulin was used as loading control. **D.** We co-transfected PC12 cells with BMP7 and scr-siRNA or siRNA-*Itgb1* and 24 h later proliferation, migration and invasion were assessed. Values for cells with knockdown of *Itgb1* were normalized against the values of scr-siRNA-transfected cells arbitrarily set to 100%. The experiments were performed two times with three technical replicates with similar results. For proliferation, the average of the 2 experiments is shown. For migration/invasion, five random fields of each test at × 400 magnification were counted (average ± SD). **, *P* < 0.01; ***, *P* < 0.001. In parallel, the levels of BMP7, integrin β1, P-AKT and AKT were assessed in the transfected cells by western blotting with specific antibodies as described above. α-Tubulin was used as a loading control. **E.** PC12 cells were treated with the indicated concentrations of the P-AKT inhibitor GSK690693 and western blot analysis was performed 24 h later to determine the expression of BMP7, integrin β1, P-AKT, and AKT. α-Tubulin was used as a loading control. **F.** Expression of BMP7, integrin β1, and P-AKT in 10 human primary PCCs and one human normal medulla (cont.) was assessed by western blotting using specific antibodies. α-Tubulin was used as a loading control.

Given that BMP7 overexpression stimulates PCC cell migration/invasion, we decided to comprehend the involved molecular mechanisms. We focused on integrins, cell surface receptors that are critical regulators of cell motility [31]. Among the integrin subunits, integrin β1 has been associated with the progression of several cancer types. As it was found expressed in all three PCC cell lines (data not shown), it was selected for further studies. If integrin β1 was involved in Bmp7-mediated increase in PC12 cell migration/invasion, we expected it to be induced by activated Bmp7 signaling. Indeed, ectopic *BMP7* overexpression increased not only PC12 cell migration/invasion (Figure 4A, 4B) but also the amount of integrin β1 (Figure 5A). As assessed by IF, *BMP7* up-regulation enhanced integrin β1 expression in transfected and nearby un-transfected cells, consistent with a paracrine signaling mechanism. In contrast, empty-vector-transfected cells exhibited extremely faint staining for integrin β1 by both IF and western blotting (Figure 5A). PC12 cells were also treated with recombinant human (rh) BMP7, which up-regulated the canonical downstream pathways (i.e., P-Smad1/5/8) similar to *BMP7* overexpression (Figure 5B). Following rhBMP7 addition to the culture media, integrin β1 expression was assessed at different time points and was found to be induced 15 min post-stimulation (Figure 5C). To verify that integrin β1 mediates the BMP7-dependent increase in cell migration and invasion, PC12 cells were co-transfected with Myc-BMP7 and scrambled siRNA oligos, or with Myc-BMP7 and siRNA oligos against the *Itgb1* gene (si*Itgb1*) encoding integrin β1. *Itgb1* silencing significantly reduced PC12 cell proliferation, migration and invasion capacity (Figure 5D). Efficient *Itgb1* knockdown was confirmed by western blotting (Figure 5D).

Similar to integrin β1, P-AKT rapidly increased in rhBMP7-treated PC12 cells (Figure 5C). Integrin β1 is regulated by PI3K/AKT/mTOR signaling in other cancers [32, 33]. Therefore, we investigated whether this also occurs in PCC. Upon incubation with the selective pan-AKT kinase inhibitor GSK690693, integrin β1 level decreased in PC12 cells with BMP7 overexpression (Figure 5E), consistent with a role for AKT in modulating integrin β1 expression. In PC12 cells, as in other cancer cells [34], P-AKT did not decrease following GSK690693 treatment because of the blockade of a negative feedback loop downstream of AKT.

As no data were available on integrin β1 expression in human PCCs, 10 tumors with enough frozen material were analyzed by western blotting and compared with normal adrenal medulla. Samples were also probed for BMP7 and P-AKT expression. The results revealed that integrin β1 is expressed in PCCs; its level tends to correlate with that of BMP7 (Figure 5F). Primary PCCs having high BMP7 and integrin β1 levels mostly showed also elevated P-AKT.

### BMP signaling inhibition suppresses PCC cell proliferation and promotes apoptosis

**Figure 6:**
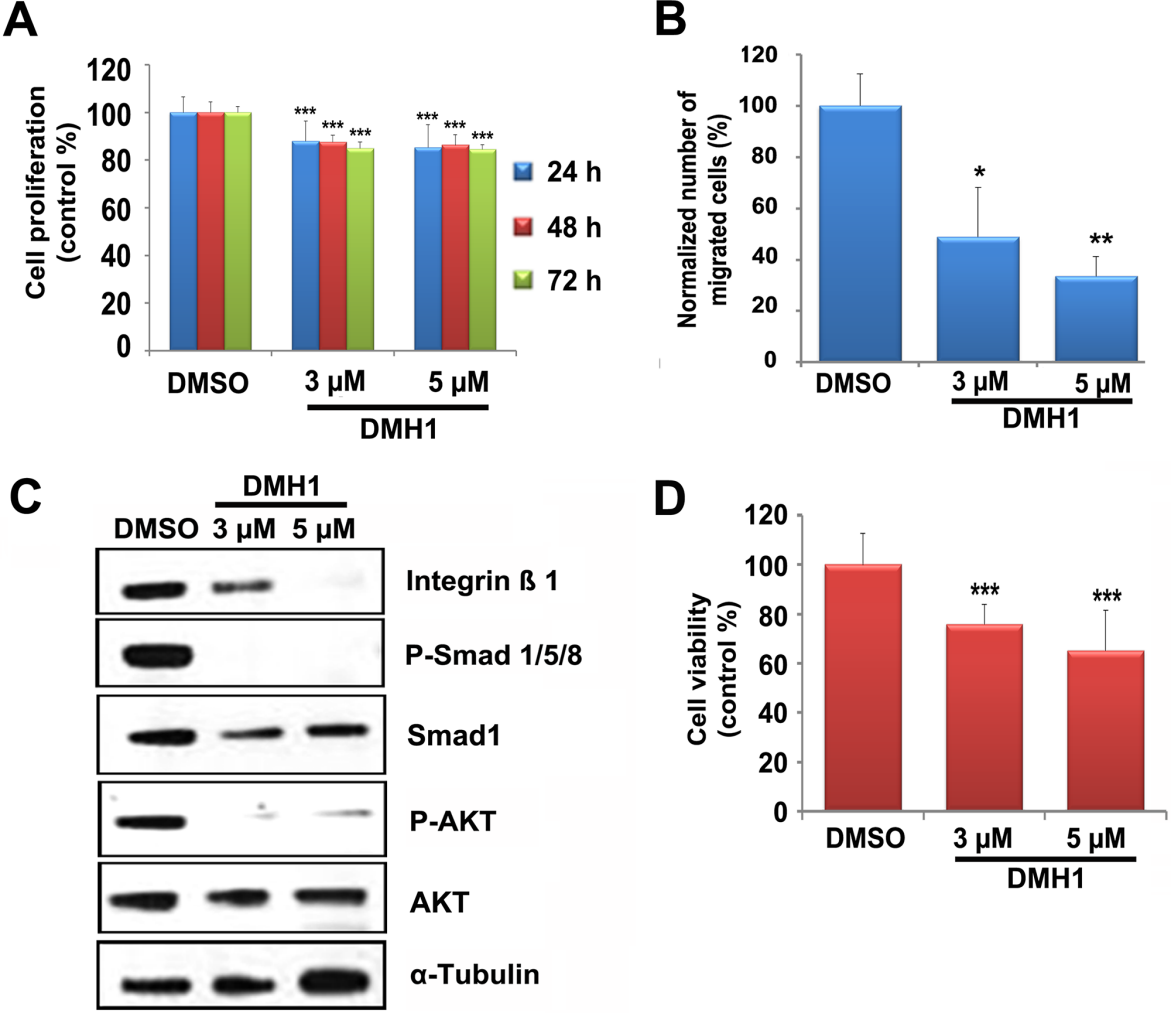
Effect of DMH1 on BMP downstream signaling and PCC cell growth **A.** MTT cells were treated with DMH1 (3 μM or 5 μM) or with DMSO vehicle. Proliferation was assessed at the indicated times by the WST-1 assay. The experiments were performed two times with six technical replicates each, and are expressed as the mean ± SD. The values are normalized against those of DMSO-treated cells set to 100%. ***, *P* < 0.001. **B.** MTT cells treated as in A for 24 h were used for migration assays as indicated in Figure 4. The percentage of cells that migrated was normalized against the values of DMSO-treated cells arbitrarily set to 100%. The experiment was performed two times with three technical replicates with similar results. Five random fields of each test at ×400 magnification were counted (±SD). *, *P* < 0.05; **, *P* < 0.01. **C.** MTT cells were treated with DMH1 (3 μM or 5 μM) or with DMSO vehicle for 24 h. Proteins were then collected and probed for the expression of integrin β1, P-Smad1/5/8, Smad1, P-AKT and AKT. α-Tubulin was used as a loading control. **D.** Rat primary tumor cells were treated with DMH1 (3 μM and 5 μM) or DMSO for 72 h and cell viability was assessed using the WST-1 assay. Shown is the average of five independent cultures from five mutant rats (8 months of age). The values are normalized against those of DMSO-treated cells set to 100%. Data were analyzed independently with six technical replicates each and are expressed as the mean ± SD. ***, *P* < 0.001.

Due to its involvement in human cancers, the BMP pathway has been considered for therapeutic intervention, and small-molecule BMP antagonists are being evaluated in preclinical studies. Among them, DMH1, a second-generation analog of dorsomorphin, highly selectively inhibits BMP type I receptors (BMPR-I), but no other off-target receptors [35]. To verify whether blocking BMP receptor signaling might be a potential strategy for targeted therapy of PCC, we treated MTT cells (high endogenous Bmp7 levels) with DMH1 and then assessed cell proliferation and migration. DMH1 treatment significantly suppressed MTT cell proliferation in a dose- and time-dependent manner (Figure 6A), and even more strongly inhibited cell migration (Figure 6B). Concomitantly, we observed a dose-dependent downregulation of the expression of P-Smad1/5/8 and integrin β1, both readouts of active BMP signaling in PCC cells, as well as of P-AKT in DMH1-treated MTT cells (Figure 6C).

Next, we determined the effect of DMH1 on rat primary PCC cells (high Bmp7 expression). Independent cultures from 5 affected rats were incubated with different DMH1 concentrations, and this resulted in a strong decrease in cell viability (Figure 6D).

In conclusion, a novel small-molecule BMP antagonist elicits anti-proliferative and anti-migratory responses in PCC cells with active BMP signaling *in vitro*.

### Tumors expressing high Bmp7 levels respond well to PI3K inhibition *in vivo*

**Figure 7:**
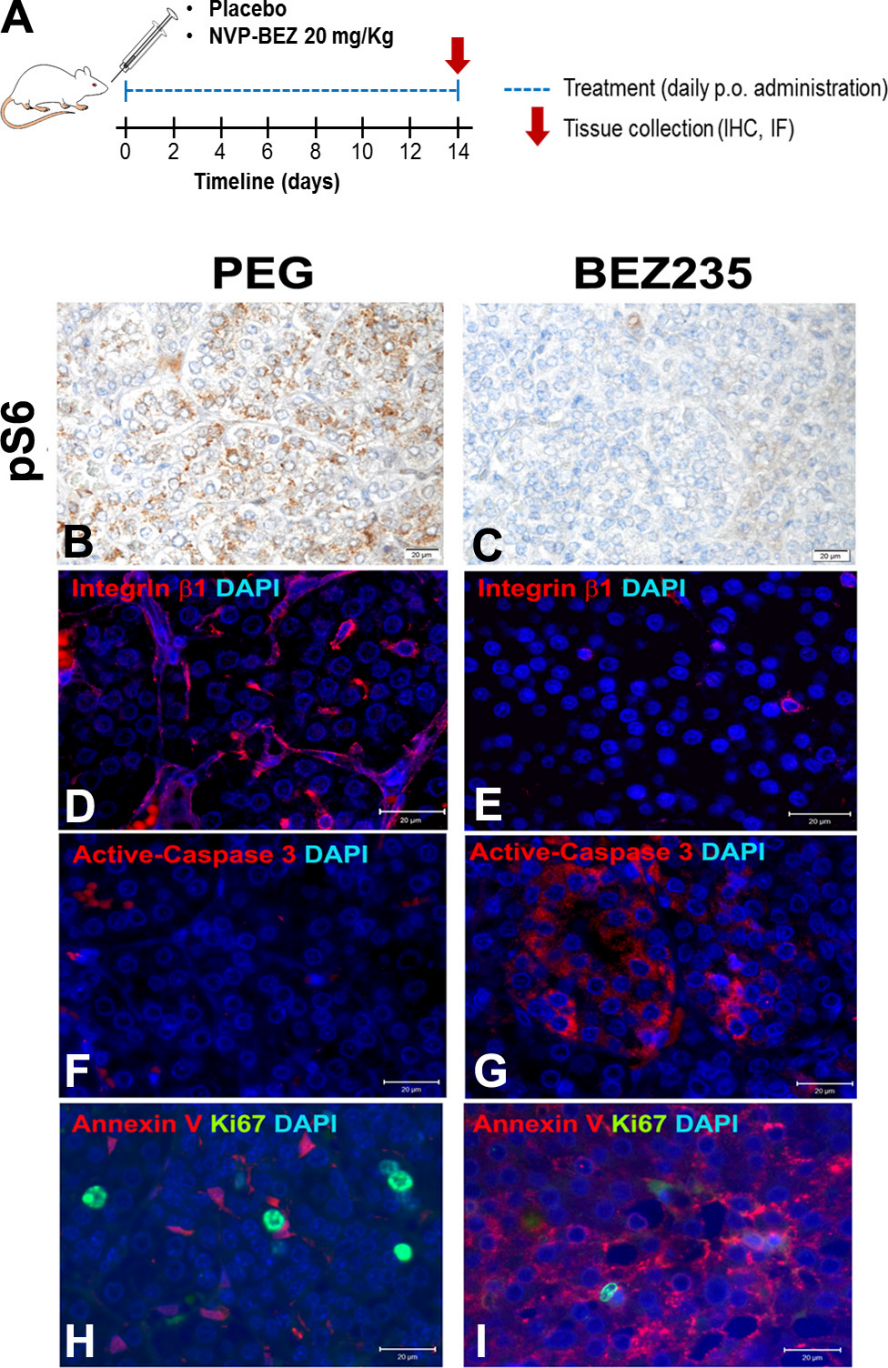
*Ex vivo* analysis of rat PCCs following placebo or NVP-BEZ235 treatment MENX-affected rats (*n* = 4) were treated with a dual PI3K/mTOR inhibitor, NVP-BEZ235, or with PEG vehicle (*n* = 4) by oral gavage for 2 weeks then adrenal glands were collected. **A–I.** IF or IHC on tissues collected from the rats was performed using specific antibodies raised against P-S6, integrin β1, Ki67, Annexin V or active-caspase3. For IHC, haematoxylin was used as counterstaining, while for IF, cell nuclei were counterstained with DAPI. Original magnification: ×400. Scale bars: 20 μM.

The increase in P-AKT following either *BMP7* gene transfection or rhBMP7 treatment suggests that BMP7 signaling occurs through the PI3K/AKT/mTOR pathway in PCC cells. Given our data on the high BMP7 levels in PCCs and on its pro-oncogenic function, targeting the PI3K pathway may represent an effective therapeutic strategy for treating these tumors. To test this hypothesis, we employed MENX rats as a prototypic *in vivo* model of endogenous PCCs having elevated BMP7 expression and an activated PI3K pathway [36]. We previously reported that NVP-BEZ235, a dual PI3K-mTOR inhibitor, has antitumor activity against primary PCC cultures from MENX rats *in vitro* [36]. Here we daily administered NVP-BEZ235 (*n* = 4) or PEG vehicle (*n* = 4) to MENX-affected rats by oral gavage for 2 weeks. Post-treatment, we observed a reduction of both P-S6 (Figure 7A, 7B), a downstream effector of mTOR, and integrin β1 (Figure 7C, 7D), readout of BMP signaling, in tumors following NVP-BEZ235 but not vehicle administration, thereby confirming BMP/PI3K pathway blockade. Concomitantly, PCCs of drug-treated rats displayed a significant reduction in proliferation (Ki67 staining) and increase in apoptosis (Annexin V and active caspase 3 staining) (Figure 7F–7I). These findings *in vivo* recapitulate the signaling pathway that we identified in PCC cells *in vitro*, wherein active BMP7 signaling induces integrin β1 expression through PI3K/AKT/mTOR pathway activation. Moreover, they demonstrate that targeting the PI3K signaling cascade evokes antitumor effects in PCCs with active BMP signaling *in vivo*.

## DISCUSSION

The molecular mechanisms accompanying sporadic PCC remain largely undefined, thereby hampering the identification of novel therapeutic targets for this occasionally aggressive and malignant tumor. In this study, we show that BMP7 is highly expressed in PCC, where it promotes pro-oncogenic features through autocrine- and paracrine-mediated activation of PI3K/AKT/mTOR signaling cascade and of integrin β1 expression. We demonstrated that this knowledge may be exploited for therapeutic purposes in proof-of-principle studies using *in vitro* and *in vivo* PCC models.

We previously reported that *BMP7* is overexpressed in human PCC [14]. In this study, we demonstrate that the BMP7 protein is highly expressed in 72% of human tumors and it associates with tumor location (more frequently elevated in PGLs than in PCCs) and tumor size (higher in large PCCs). Of note, tumor size positively correlates with a higher malignancy risk for PCC [6], and, although data are not always consistent, an increased malignancy rate has been reported for PGLs compared with PCCs [6]. In our patient cohort, nine of 11 malignant tumors (82%) had high BMP7 expression. As BMP7 has already been shown to be a marker of tumor progression and metastases development in breast and esophageal cancers [25, 37], it is worth further exploring the potential of BMP7 as a marker of malignant behavior by analyzing additional malignant PCC cases for its expression.

Our understanding of the contribution of BMP signaling to cancer biology remains limited. Although activation of BMP-mediated pathways inhibits cell growth and induces apoptosis in various cancer cell types, it can also be implicated in increasing metastatic potential and tumor angiogenesis in other tumor types [25, 26, 38]. To date, the contribution of BMP7 signaling to PCC pathogenesis has not been addressed. By manipulating endogenous *Bmp7* levels in PCC cell lines (overexpression and knockdown) and in primary rat tumor cells (knockdown), we could demonstrate that endogenous *Bmp7* overexpression promotes oncogenic features in these cells. Specifically, high *Bmp7* gene expression promoted the proliferation and potently induced the migration and invasion of PC12, MPC, and MTT cells. This situation is reminiscent of what has been reported about activation of BMP2- and BMP4-dependent pathways in other tumor types [39, 40]. Moreover, shRNA-mediated *Bmp7* gene silencing reduced the viability of rat primary PCC cells, suggesting that in this experimental system Bmp7 sustains cell survival. Thus, similar to what has been reported for lung cancer [41], melanoma [42] and, albeit controversial, for breast cancer [25], BMP7 exerts a tumor-promoting action in PCC.

Because BMP7 strongly increases the motility and invasion of PCC cells, we investigated the molecular mechanisms that might mediate these effects. We focused on integrin β1: a major cell adhesion molecule in mammalian cells, extensively associated with the motility and invasion of tumor cells, and expressed in all PCC cell lines. No data were available up to now on integrin β1 involvement in PCC. We observed that both ectopic *Bmp7* overexpression and treatment with rhBMP7 associate with enhanced integrin β1 expression in PC12 cells, whereas selective blockade of BMP signaling by DMH1 causes a drastic reduction in integrin β1 expression in MTT cells. Knockdown of *Itgb1* (integrin β1) suppresses BMP7-mediated increase in proliferation, migration and invasion of PC12 cells. These findings provide experimental evidence to support the functional link between the BMP pathway and integrin β1. Moreover, analysis of human primary PCCs revealed for the first time that integrin β1 is highly expressed in these tumors and its amount tends to correlate with that of BMP7. In breast cancer cells, it has been reported that BMP7 inhibits TGFbeta-induced invasion via inhibition of integrin β3 [43], again attesting to the cell-type dependent effect of BMP7 signaling. In conclusion, we identified integrin β1 as a downstream target of BMP-dependent signaling in PCC and as a tumor progression driver.

Human primary PCCs are characterized by the hyperactivation of the PI3K/AKT pathway [1, 44]. Here we report that over half of PCCs are characterized by high BMP7 expression, and that active BMP-dependent pathways increase P-AKT levels in PCC cells. Thus, it is tempting to speculate that the PI3K pathway activation observed in PCCs might be in part due to BMP7 expression upregulation and the triggering of a cell autonomous stimulatory pathway. Mechanistically, we postulate that BMP7 signaling activates the PI3K/AKT/mTOR pathway, which in turn leads to the up-regulation of integrin β1. Indeed, inhibiting AKT or PI3K/mTOR activities in the context of active BMP7-mediated pathways reduced integrin β1 expression in PCC both *in vitro* and *in vivo*. Integrin β1 up-regulation by the activated PI3K/AKT pathway has been also documented in other cancers, including breast [32] and prostate carcinomas [45], where it accounts for enhanced cell motility and invasion.

Our data uncover downstream effectors of active BMP signaling that could be the target of novel therapeutic strategies for PCC. To verify this, we investigated whether the inhibition of the PI3K/AKT/mTOR pathway is effective against PCCs expressing high levels of BMP7. Specifically, we exploited our MENX rat model to study the response of endogenous PCCs to a dual PI3K/mTOR inhibitor (NVP-BEZ235) that is bioavailable and in clinical trials. NVP-BEZ235 displays antitumor effects against PCC cell lines and primary rat PCC cells *in vitro* [36, 46]. Here, *ex vivo* analysis of the rat tumors confirmed that NVP-BEZ235 inhibits BMP and PI3K/mTOR signaling, reduces integrin β1 expression and cell proliferation, and induces cell death. Collectively, these data support our hypothesis that targeting the PI3K signaling cascade holds promise in PCC treatment as a paradigm of tumors with active BMP7-mediated signaling. They also suggest that BMP7 might be a potential predictor of response to such antitumor agents.

To date, everolimus (rapamycin analogue) was evaluated in a few patients with progressive/malignant PCCs, but exhibited low efficacy [10, 11]. This lack of tumor control could be due to the activation of a feedback loop reactivating AKT signaling upstream of mTOR, a well-documented mechanism of resistance to rapamycin and its analogues in various human cancers [47, 48]. To escape the feedback resistance, compounds able to inhibit both mTOR and the upstream PI3K kinase were generated, including NVP-BEZ235 [49]. Our preclinical *in vivo* trial provides the rationale for targeting the PI3K pathway in patients with progressive/inoperable PCCs using dual PI3K/mTOR inhibitors instead of single-molecule inhibitors.

While drugs inhibiting the PI3K pathway have reached the clinical trial stage or are already approved for treatment, BMP antagonists are still in preclinical development, but may be available for clinical applications in the future. Among them is DMH1, the most selective small molecule inhibitor of BMP receptor signaling currently available [35]. DMH1 inhibits the intracellular kinase domains of BMPR-I molecules such as ALK1 (activing receptor-like kinase 1), ALK2, ALK3/BMPR-IA and ALK6/BMPR-IB, but does not inhibit ALK4 and ALK5, nor other kinases such as AMP-activated kinase, VEGF (vascular endothelial growth factor) receptor type 2 and PDGF (platelet-derived growth factor) receptor β [35, 50]. DMH1 has recently been shown to inhibit breast-to-lung metastases and to reduce primary growth of mammary carcinomas in mice [50], and to suppress the tumor growth of a human lung cancer xenograft model [51]. Our data of an antitumor effect elicited by DMH1 in PCC cells hold great promise for future targeted therapies of these tumors.

In summary, our work identifies BMP7 as a novel pro-oncogenic factor in PCC and provides leads for novel therapeutic approaches against PCCs targeting downstream effectors of BMP7-mediated signaling.

## MATERIALS AND METHODS

### Human pheochromocytoma samples

This study was approved by the Ethics Committees of the Universities of Munich, Tübingen, and Florence; informed written consent was obtained from all patients. PCC tissues were snap-frozen after surgery and stored at −80°C until required. Frozen normal adrenals and tumors were also provided by the Imperial College Healthcare NHS Trust Tissue Bank (Project R13041).

Tissue microarrays (TMAs) containing 208 human adrenal tumors (166 PCCs, 42 PGLs) and control tissues were obtained from the ENS@T (European Network for the Study of Adrenal Tumors) and previously described [52]. For each tumor, two different areas (cores) were selected for the arrays.

### Rat tissue and plasma samples

Adrenals of age-matched wild-type and MENX-affected rats were fixed in 4% buffered formalin and embedded in paraffin. Histological examination confirmed the diagnosis of PCC. Three-micrometer sections were used for IHC or IF. Plasma from age-matched wild-type and MENX-affected rats was obtained at autopsy and stored at −80°C. Plasma Bmp7 levels were measured with an ELISA-KIT obtained from USCN Life Science Inc. (Wuhan, China).

### Cell culture, treatments, and assays

Mouse PCC cells, MPC 4/30 PRR [53], derived from a heterozygous Nf1-mutant mouse PCC, and its more aggressive derivative MTT [54] cells, were kindly provided by Dr. Karel Pacak (NIH, Bethesda, Maryland, USA) and cultured in DMEM medium (Sigma, Hamburg, Germany) containing 10-ml fetal bovine serum (FBS) and 1% (v/v) penicillin/streptomycin at 37°C in a 5% CO_2_ atmosphere. Rat PC12 cells were purchased from LGC Standards (Wesel, Germany) and cultivated, following manufacturer's instructions. All cells were routinely tested for mycoplasma and found free of contamination. Moreover, they were maintained in culture for maximum 5 passages after thawing. Primary PCC cells from MENX mutant rats were established and cultivated as previously reported [36].

PC12 cells were transfected with plasmid DNA as described [17], or with 200 pmol of scrambled (ON TARGETplus Non-targeting siRNA, Dharmacon, Lafayette, CO, USA) or pooled siRNA against rat *Itgb1* gene (ON-TARGET plus SMART pool siRNA, Dharmacon) by Amaxa 4D-Nucleofector (Lonza) following the manufacturer's instruction. For infections, MPC, MTT, and primary tumor cells were plated on 96-well plates and were infected with lentiviral vectors expressing either green fluorescence protein (GFP) alone (pGreenPuro #SI505A-1-SBI, BioCat, Heidelberg, Germany) or GFP and shRNA against *BMP7*. Virus productions were performed as published [55] and infections slightly adjusted to the described protocol. Two different shBmp7 sequences were originally cloned into a pGreenPuro lentiviral vector: #1.8: FW AGGCCTGATTGGACGGCAT; #2.9: FW 5′-CGGGA GAUGCAGCGGGAAA-3′.

WST-1 colorimetric assay (Roche, Mannheim, Germany) for cell viability was performed 72 h after infection according to the manufacturer's recommendations.

### Protein extraction and western blotting

Cells were harvested after treatment with rhBMP7 transfection or infection at specific timepoints. Total proteins were extracted from cells or frozen tissues, and western blotting was performed as previously reported [56]. The primary antibodies used are: human anti-BMP7 (R&D Systems, USA; clone #164311; dilution 1:1000); rat anti-Bmp7 (#4693, 1:100), P-Smad1/5/8 (#9511, 1:50), Smad1 (#5753; 1:500), AKT (#9272; 1:500) and P-AKT (#9271; 1:500) all from Cell Signaling Technology (Danvers MA, USA); integrin β1 (Abcam, Cambridge, UK; #EP1041Y; dilution 1:500); anti-Myc tag (Clontech, St-Germain-en-Laye, France; #631206; dilution 1:500), α-Tubulin (Sigma-Aldrich, Hamburg, Germany; #T5168; dilution 1:1000). Western blotting experiments from biological replicates showed similar expression data, attesting to the reproducibility of the results.

### IHC and IF

IHC was performed on an automated immunostainer (Ventana Medical Systems, Tucson, AZ) as previously described [57]. The primary antibodies used are: rat anti-BMP7 antibody (see above; dilution 1:100), human anti-BMP7 (see above; dilution 1:1000), anti-P-S6 antibody (Cell Signaling Technology; #2211; 1:500). Primary antibodies were diluted in Dako REALTM antibody diluent (Dako, Hamburg, Germany). Haematoxylin was used as counterstaining. Positive controls were included in each batch. TMAs were scored for BMP7 expression using a semi-quantitative method taking into account the staining intensity: − (negative), + (mild), ++ (moderate), and +++ (strong). Slides were scored using a double-blind method by two independent observers; the percentage of discrepancies was below 3%. We only considered tumors for which both cores could be scored after IHC staining processes (inclusion criteria). Images were recorded using a Hitachi camera HW/C20 installed in a Zeiss Axioplan microscope with Intellicam software (Carl Zeiss MicroImaging, Göttingen, Germany).

For IF, we used primary antibodies against BMP7 (see above), P-Smad1/5/8 (see above), integrin β1 (1:200), Ki67 (BD Biosciences, Heidelberg, Germany; #550609; 1:100), Annexin V (Abcam; ab14196; 1:100) or active-caspase3 (Cell Signaling; #9664; 1:200). Cell nuclei were counterstained with DAPI. Secondary anti-mouse Alexa Fluor^®^ 555 Conjugate (Cell Signaling), secondary anti-rabbit FITC-conjugated antibody (Invitrogen, Darmstadt, Germany) were used as reported [58]. Sections were analyzed with a Zeiss Axiovert 200 epifluorescence microscope including Apotome unit (Carl Zeiss).

### Migration and invasion assays

Chemomigration assays were performed using 24-well plates with uncoated polycarbonate membrane inserts (BD BioCoat™, BD, Heidelberg, Germany). Cells were transfected with Myc-BMP7 or empty vector, and trypsinized and collected 24 h later in a medium containing 0.02% FBS and 0.1% horse serum (HS). A total of 50000 cells (PC12) or 100000 cells (MPC/MTT) were added onto the insert. The lower well was filled with a medium complemented with 2.5% FBS and 15% HS. Migrated cells were fixed 24 h later in 100% methanol and stained with 1.5% (w/v) toluidine blue in water. MPC and MTT cells were infected for *Bmp7* knockdown and the same procedure as above was employed after 72 h. Invasion assays were performed with matrigel-coated polycarbonate membrane inserts (BD BioCoat™, BD) according to the manufacturer's recommendations. We plated 75000 cells (PC12) or 150000 cells (MPC/MTT) for these assays.

### Quantitative TaqMan RT-PCR

RNA was extracted using RNeasy Mini Kit (Qiagen, Hilden, Germany) following the manufacturer's instructions. Quantitative RT-PCR was performed using TaqMan inventoried primers and probes (Applied Biosystem, CA, USA) for the indicated genes, following previously reported protocols [14].

### RT-PCR analysis of Bmp receptor genes

Semiquantitative RT-PCR was performed for Bmp receptor transcripts using RNA extracted from microdissected adrenomedullary cells of the tissue samples or MPC, MTT, PC12, and primary cells of rat PCCs. Primers used for semi-quantitative analysis are described in Supplementary Table S1. Conditions for the RT-PCR reaction were as previously reported [14].

### *In vivo* treatment

This study was approved by the ethics committee on animal research of the government of Upper Bavaria, Germany. MENX-affected rats were maintained as previously reported [12] in agreement with the procedures approved by the Helmholtz Zentrum München, by the Technische Universität München, and by the local government authorities.

NVP-BEZ235 was kindly supplied by Novartis Pharma. In preliminary experiments, various doses of NVP-BEZ235 were tested in MENX rats, as reported [58], and the dose of 20 mg/Kg was selected. MENX-affected rats at 7–8 months of age (with adrenal tumors but still in good general health) were treated for 14 days with NVP-BEZ235 (20 mg/kg) or placebo (PEG) administered daily per oral gavage. At the end of the treatment, adrenal glands were collected for IHC and IF of relevant markers.

### Statistical analyzes

Study endpoints from the *in vitro* experiments, including cell proliferation, migration, and invasion, as well as primary cell viability are summarized using bar graphs with means ± SD. A paired two-tailed Student's *t* test was used to detect significance between two series of data, and *P* < 0.05 was considered significant. For TMA results, the staining results were compared with the clinicopathological features and correlations were assessed using Fisher's exact test.

## SUPPLEMENTARY TABLE AND FIGURES


