# Supplementary material for: Oncogenic features of the bone morphogenic protein 7 (BMP7) in pheochromocytoma
Source: Oncotarget. 2015 Aug 18;6(36):39111–26. doi: 10.18632/oncotarget.4912 (PMC4770760; doi:10.18632/oncotarget.4912)
Supplement: Supplementary file 1 [file oncotarget-06-39111-s001.pdf]

SUPPLEMENTARY TABLE AND FIGURES

Supplementary Table S1: Primer sequences used to amplify the genes encoding the BMP7 receptors in rat and mouse PCC cells and in rat tissues

Rat		Mouse	
rAcvr2b-F	5′-CATCACGTGGAACGAACTGTGC-3	*mAcvr2a*-F	5′-AAACGGCGACATTGTTTTGC-3
rAcvr2b-R	5′-AGCATGTACTCATCGACAGGCC-3	*mAcvr2a*-R	5′-GTGTGACTTCCATCTCCGGA-3
rAcvr2a-F	5′-TCAGACTGGTGTTGAGCCTT-3	*mAcvr2b*-F	5′-CATCACGTGGAACGAACTGTGC-3
rAcvr2a-R	5′-GTGTGACTTCCATCTCCGGA-3	*mAcvr2b*-R	5′-AGCATGTACTCATCGACAGGCC-3
rAlk2-F:	5′-TGCTAATGATGATGGCTCTCC-3	*mAlk2-F*:	5′-TCGATGGAGTAATGATCCTTCC-3
rAlk2-R:	5′-CGATCCAGGGAAGGATTTC-3	*mAlk2-R*:	5′-CCGTGATGTTCCTGTTACACC-3
rAlk3-F:	5′-GGAGGAATCGTGGAGGAATAT-3	*mAlk3-F*:	5′-CAGACTTGGACCAGAAGAAGCC-3
rAlk3-R:	ATACGCAAAGAACAGCATGTC-3	*mAlk3-R*:	5′-ACATTCTATTGTCTGCGTAGC-3
rAlk6-F:	5′-AGACTGAGATATATCAGACGGTCC-3	*mAlk6-F*:	5′-AGACTGAGATATATCAGACGGTCC-3
rAlk6-R:	5′-CGTGTGTAGATGGCACAGG-3	*mAlk6-R*:	5′-CGTGTGTAGATGGCACAGG-3
rBmpr2-F:	5′-GGAGAAATCAAAAGGGGAC-3	*mBmpr2-F*:	5′-GGAGAAATCAAAAGGGGAC-3
rBmpR2-R:	5′-CTCCTGTCAACATTCTGTATCC-3	*mBmpR2-R*:	5′-CTCCTGTCAACATTCTGTATCC-3

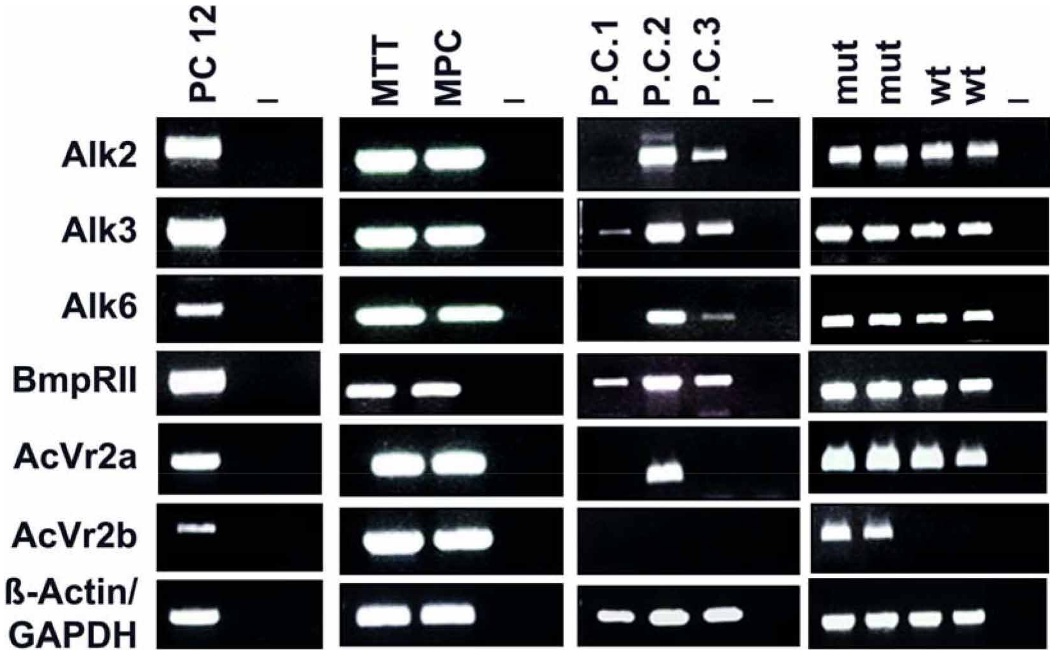

Supplementary Figure S1: Bmp7 receptor expression in PCC cells and tissues. RNA was extracted from three rat PCC primary cell cultures (P.C. #1–3), from PC12, MPC and MTT cells and from adrenal medullary tumors of two mutants (mut) and from adrenal medulla of two wild-type (wt) rats. RNA was reverse transcribed into cDNA, and amplified by RT-PCR using primers specific for Alk 2, 3, and 6, BmpRII, AcVr2a,b, ß-Actin (for rat), and GAPDH (for mouse). The PCR products were detected on a 1% agarose gel following ethidium bromide staining.

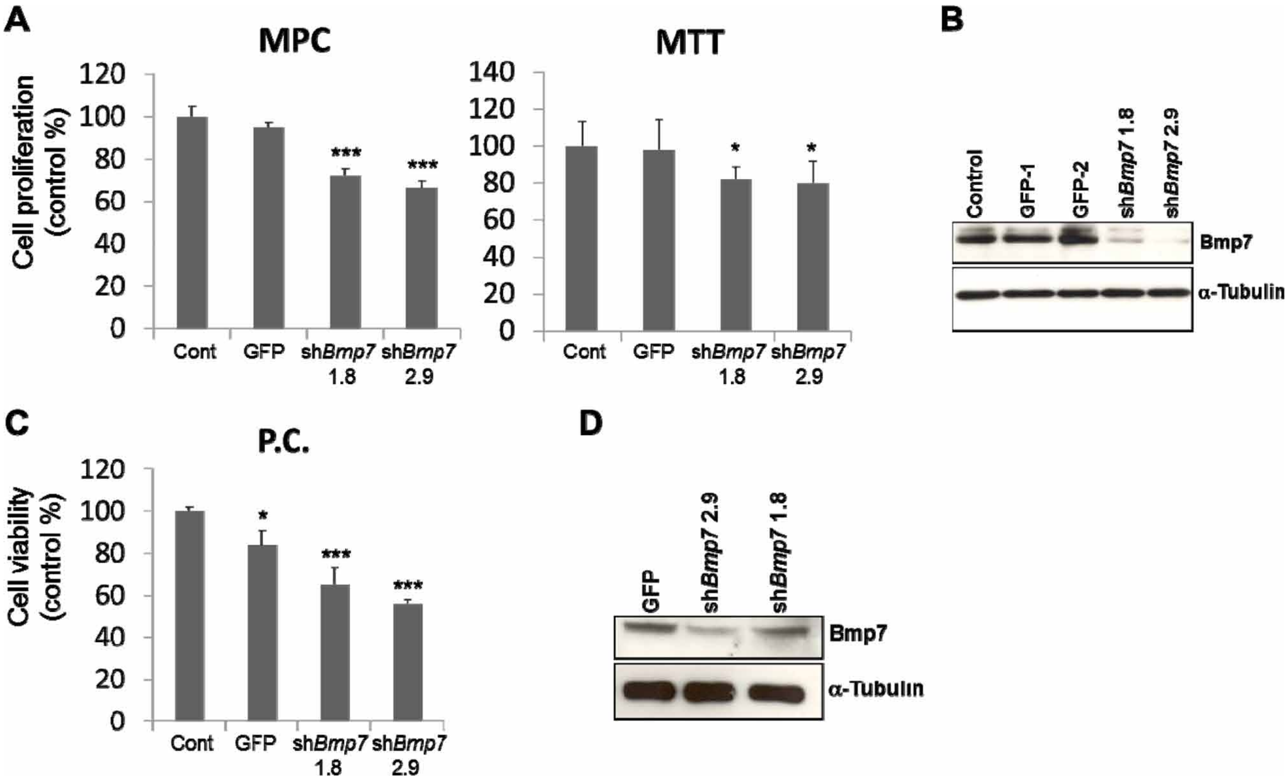

Supplementary Figure S2: shRNA-mediated knockdown of *Bmp7* in PCC cell lines and primary cells. A. MPC and MTT cells were infected with lentiviral vectors containing sh*Bmp7*-GFP (#1.8, #2.9), GFP alone (GFP) or left uninfected (Cont), and 72 h later we assessed proliferation using the WST-1 assay. Data were analyzed independently with six technical replicates each, and are expressed as the mean ± SD. Proliferation was normalized against the values of uninfected cells arbitrarily set to 100%. *, $P < 0.05$; ***, $P < 0.001$ **B.** In parallel to A, total proteins were extracted from MPC cells and western blotting was performed using the anti-BMP7 antibody. α-Tubulin was used as loading control. **C.** Primary rat PCC cells (P.C.) were infected with lentiviral vectors containing sh*Bmp7*-GFP (#1.8, #2.9), GFP alone (GFP) or left uninfected (Cont) and cell viability was measured 72 h after infection. The average of two independent cultures from two mutant rats is shown. Proliferation was normalized against the values of uninfected cells which was arbitrarily set to 100%. *, $P < 0.05$; ***, $P < 0.001$. **D.** From infected primary PCC cells treated as in C, proteins were extracted and western blotting was performed using the anti-BMP7 antibody. α-Tubulin was used as loading control.
